# Permissiveness of American bison to infection with severe acute respiratory syndrome coronavirus-2

**DOI:** 10.1038/s41598-025-10100-3

**Published:** 2025-09-29

**Authors:** Mitchell V. Palmer, Alexandra Buckley, Eric D. Cassmann, Steven C. Olsen, Daniel W. Nielsen, Ellie J. Putz, Hannah Seger, Jeffrey C. Chandler, Paola M. Boggiatto

**Affiliations:** 1 National Animal Disease Center, Agricultural Research Service, USDA, 1920 Dayton Ave, Ames, IA 50010 USA; 2 National Wildlife Research Center, Animal and Plant Health Inspection Service, USDA, Ft. Collins, CO USA; 3 Oak Ridge Institute for Science and Education, Oak Ridge, TN USA

**Keywords:** *Bison bison*, Coronavirus, Intranasal, SARS-CoV-2, Microbiology, Zoology

## Abstract

The COVID-19 pandemic represents one of the most significant public health events of the last century. As with other coronaviruses (SARS, MERS) the role of animals is of intense interest. Believed to have originated in bats, the role of other animals in the epidemiology of the SARS-CoV-2 pandemic is still unclear, as is the range of susceptible hosts. American bison were intranasally infected with SARS-CoV-2 and monitored for seroconversion and the presence of viral RNA in oronasal secretions and feces. Although clinical signs were not seen, permissiveness of bison to infection with SARS-CoV-2 was manifest by seroconversion, the presence of viral RNA in oronasal secretions, persistence of viral RNA in lymphoid tissue, and viral associated interstitial pneumonia. Retrospective sequencing of the inoculum revealed a common in vitro adaptation in the furin cleavage site of the spike protein that may have reduced in vivo viral fitness. As such, we cannot exclude the possibility that use of an isolate with an intact furin cleavage motif would more efficiently infect bison.

## Introduction

Severe acute respiratory syndrome coronavirus 2 (SARS-CoV-2), a novel coronavirus, is the cause of coronavirus disease 19 (COVID-19) in humans. Originating in Wuhan, China in December 2019, SARS-CoV-2 resulted in a worldwide pandemic causing over 775 million cases of COVID-19 and over 7 million deaths (https://covid19.who.int/). Believed to have originated in bats, it is hypothesized that other unidentified animal host(s) served as possible intermediaries between bats and humans. However, a specific intermediate host has not been identified. A recent analysis of environmental qPCR and sequencing data collected from the Huanan market suggests that likely intermediate hosts include raccoon dogs (*Nyctereutes procyonoides*), masked palm civets (*Paguma larvata*) and hoary bamboo rats (*Rhizomys pruinosis*)^1^.

Case reports and serological surveys have identified a number of wild and domestic animal species naturally susceptible to infection with SARS-CoV-2, albeit to varying degrees. These include American mink (*Neovison vison*)^2^, rats (*Rattus norvegicus*)^3^, otters (*Lutra lutra*)^4^, ferrets (*Mustela putorius furo*)^5^, Syrian hamsters^6^, gorillas^7^, cats, dogs^8^, lions, tigers^9^, fallow deer (*Dama dama*), red deer (*Cervus elaphus*)^10^, and white-tailed deer (*Odocoileus virginianus*) ^11–13^; however, the complete range of susceptible domestic animals and wildlife is still unclear. Of concern is the possibility of animals becoming a reservoir of SARS-CoV-2 where new variants could arise and be transmitted back to humans. Of all these susceptible species, it is believed that only white-tailed deer in North America represent a wildlife reservoir of SARS-CoV-2, however on-going research strives to identify if others exist.

SARS-CoV-2 infection involves binding of the viral spike (S) protein to the host angiotensin-converting enzyme 2 (ACE2) receptor^14^. The specificity of the interaction between virus and receptor is believed to reflect the range of susceptible hosts^15^. In one analysis, all species within the order Artiodactyla, except American bison (*Bison bison*), sheep (*Ovis aries*), and swine (*Sus scrofa*) had more than 80% probability of SARS-CoV-2 cell entry via the ACE2 receptor^16^. In a separate study examining the predicted ACE2 receptor-S binding affinity, bison were similar in binding probability to cattle, cats, and white-tailed deer^17^. Separately, based on a logistic regression model constructed using the spike binding parameters of ACE2, bison had a 0.0036 probability of virus entry, a lower probability than that predicted for domestic cattle (0.917)^16^. Further investigation found that ACE2 proteins from Primates, Bovidae and Cricetidae should recognize the receptor binding domain (RBD) of SARS-CoV-2 and matched more amino acids than Suidae ACE2^18^.

Notwithstanding these predictions, subsequent experimental studies showed that cattle and swine had a low susceptibility to SARS-CoV-2 (ancestral B.1 lineage) with limited viral replication and limited seroconversion ^19–24^. However, when more recent variants were examined, cattle were more permissive to infection with the Delta variant than the Omicron variant^23^. Serological surveys of cattle in several different regions of the world have demonstrated either an absence of seropositivity or a low level of seropositivity ^25–32^.

American bison have not been examined experimentally for susceptibility to SARS-CoV-2 infection. Surveillance of captive and free-ranging bison showed as much as 14% of bison had neutralizing antibody titers^33^. Bison represent both a wildlife species and an important agricultural commodity as alternative livestock. In North America, bison commercially raised for meat are considered livestock, meanwhile bison found on public lands are considered wildlife. Bison represent an important religious and cultural symbol among many North American indigenous peoples and are the National Mammal of the United States^34^. Once found in numbers greater than 60 million across North America^35^, indiscriminate killing, habitat loss, human encroachment, and disease had a devastating effect on their numbers during the nineteenth century^36^.

Several important diseases affect farmed and free-ranging bison in North America, including mycoplasmosis, anthrax, malignant catarrhal fever, tuberculosis, brucellosis, and paratuberculosis^37–43^. The objective of the present study was to determine the susceptibility of bison to experimental infection with SARS-CoV-2. Although one of several variants could have been used for the challenge inoculum, an ancestral variant was chosen as it was believed to be less human adapted than more recent variants, such as Delta or Omicron.

## Results

### Viral RNA detection in nasal, oral, and rectal swabs

Nasal, oral and rectal swabs were collected on days 0-, 2-, -3, 4-, 5-, 7-, 10-, 14-, and 21-days post-infection (p.i.) and analyzed for the presence of viral RNA by RT-qPCR. Viral RNA was most frequently detected on nasal swabs, with positive swabs from all bison on days 2 or 3 p.i., and from one bison (#9) on day 5 p.i. (Table 1). All but one bison (#311) had viral RNA on oral swabs from at least one time point between days 2 to 5 p.i. (Table 2). No viral RNA was detected on nasal or oral swabs after day 5 p.i. and at no time point was viral RNA detected on rectal swabs. Viral RNA was not detected at any time from nasal, oral or rectal swabs of non-infected control bison.

**Table 1:**
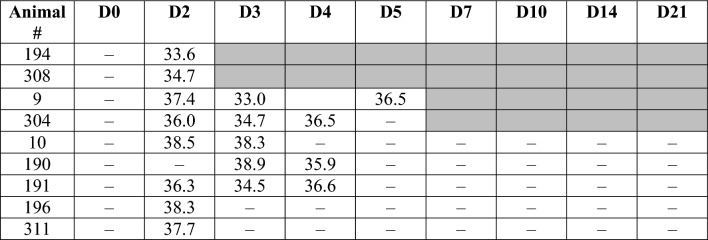
Viral RNA detection via RT-qPCR from nasal swabs collected at various times post-infection with SARS-CoV-2.

Shown are cycle threshold (Ct) values. D = day post-infection, – = not detected, gray = not done, animal euthanized.

**Table 2:**
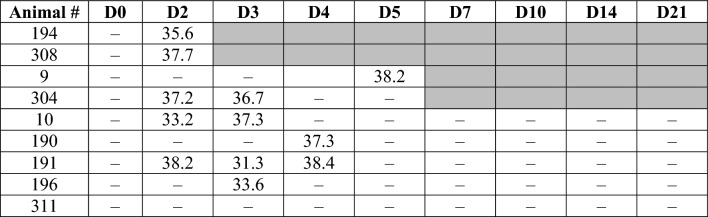
Viral RNA detection via RT-qPCR from oral swabs collected at various times post-infection with SARS-CoV-2.

Shown are cycle threshold (Ct) values. D = day post-infection, – = not detected, gray = not done, animal euthanized.

### Development of neutralizing antibody responses against SARS-CoV-2

**Fig. 1:**
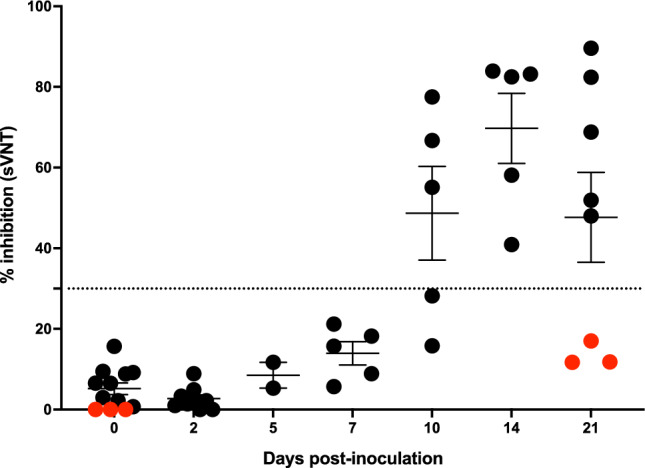
Presence of SARS-CoV-2 neutralizing antibodies in serum from bison examined by the sVNT at various time points after intranasal inoculation with SARS-CoV-2. Data are represented as mean percent inhibition ± SEM. Dotted line represents assay cut-off for positive results. Red circles correspond to results from non-infected control bison.

Serum samples were collected on days 0, 2, 5, 7, 10, 14 and 21 days p.i. to evaluate the development of SARS-CoV-2 neutralizing antibodies. Using the surrogate virus neutralization test (sVNT), neutralizing antibodies were detected as early as 10 days p.i. in 3 of 5 bison and by 14 days p.i. all bison had neutralizing antibodies, which were sustained through day 21 p.i. (Fig. 1). Virus neutralizing (VN) assays were also performed showing peak titers of 1:16 to 1:128 at days 10–21 p.i. (Table 3). Serum samples collected prior to the experiment and at necropsy of the non-infected control bison did not show evidence of seroconversion.

**Table 3:**
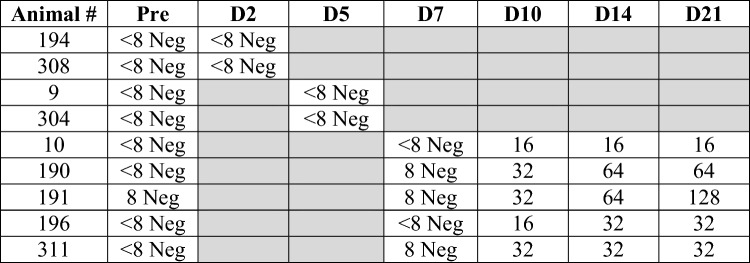
Virus neutralization (VN) titer results for bison prior to and following inoculation with SARS-CoV-2.

Pre, samples collected prior to challenge; D, day post-infection; Neg, negative, no titer.

### Gross and microscopic lesions

Both bison (#194, #308) examined at day 2 p.i. had focal to multifocal areas of congestion and hemorrhage < 1.5 cm in size limited to the right cranial lobe (#194) or the left and right caudal lobes (#308). One of the bison (#304) examined at day 5 p.i. had similar lesions in the right and left cranial lobes and right caudal lobe. Medial retropharyngeal lymph nodes of all bison examined at days 2 and 5 p.i. were characterized by multifocal to diffuse congestion and petechia. Gross lesions were not seen in any bison examined at day 21 p.i. or in non-infected control bison.

**Fig. 2:**
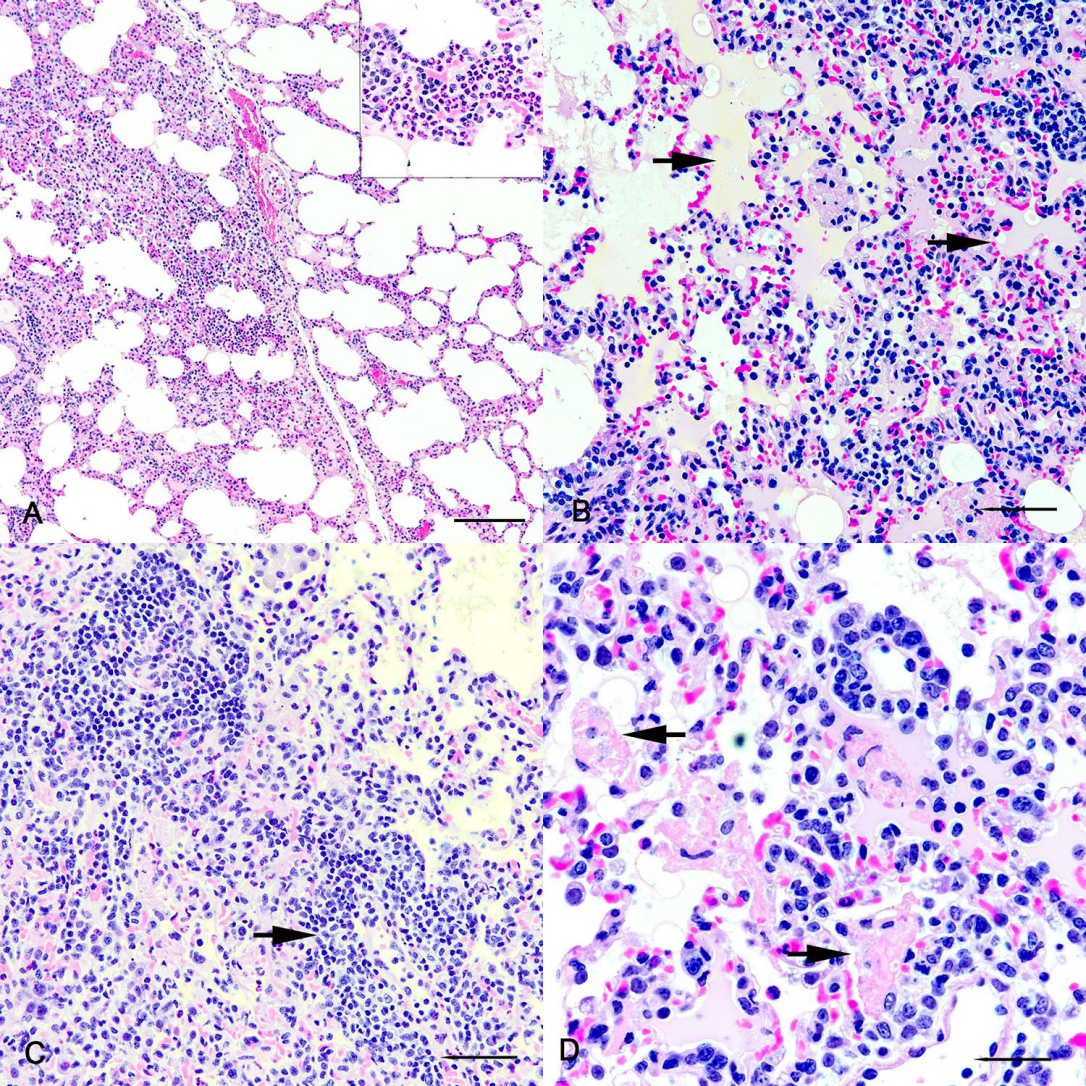
Photomicrographs of lung from bison #304 intranasally infected with SARS-CoV-2 and examined 5 days later. (**A**) Note expanded alveolar interstitium in left side of image. Multifocally, interstitial infiltrates included numerous neutrophils (inset). Bar = 200 µm. (**B**) Amphophilic to eosinophilic proteinaceous material (arrows) fills multiple alveolar lumens consistent with pulmonary edema. Bar = 100 µm. (**C**) Note perivascular infiltrates of lymphocytes (arrow). Bar = 100 µm. (**D**) Some alveolar lumens contain irregular aggregates of fibrillar and flocculent eosinophilic material consistent with fibrin (arrows). Bar = 20 µm. H&E.

Microscopically, in bison #304 examined at day 5 p.i. there were multifocal areas in the lung of expanded alveolar interstitium due to infiltrates of lymphocytes, neutrophils and macrophages with multifocal hemorrhages and free erythrocytes in alveolar lumens (Fig. 2A). Within effected areas, homogenous eosinophilic to amphophilic proteinaceous fluid (pulmonary edema) filled multiple alveoli, which also contained irregular aggregates of flocculent and fibrillar eosinophilic material consistent with fibrin (Fig. 2B, D). Some alveoli were lined by cuboidal type II pneumocytes (type II hyperplasia). Within regions of cellular infiltrate multiple vessels were surrounded by increased numbers of lymphocytes (Fig. 2C). Microscopic lesions were not seen in other infected bison or in non-infected control bison.

### Viral RNA detection by RT-qPCR in lymphoid tissues

The selection of tissues examined for the presence of viral RNA was based on previous studies using cattle, white-tailed deer, and elk^20,44–46^. Viral RNA was detected by RT-qPCR in medial retropharyngeal lymph nodes from all animals at all time points. At day 2 p.i., viral RNA was also detected in palatine tonsils and lung (Table 4). Palatine tonsils were positive for viral RNA in a single animal at 2, 5 and 21 days p.i. At no time point beyond day 2 p.i. was viral RNA detected in samples of lung. Similarly, viral RNA was not detected in any tissues examined (lung, tonsil, medial retropharyngeal lymph nodes) from non-infected control bison.

**Table 4:** Viral RNA detection via RT-qPCR in tissues from bison collected at 2-, 5-, and 21-days p.i. with SARS-CoV-2.

Animal #	PID	Right caudal lung	Left caudal lung	mRPLN	Palatine tonsil
194	2	36.7/37.2	ND	24.2/24.3	30.8/30.8
308	2	35.6/37.4	ND	31.0/31.0	ND
9	5	ND	ND	34.2/34.5	38.7/–
304	5	ND	ND	27.2./27.3	ND
10	21	ND	ND	28.2/28.4	38.6/–
190	21	ND	ND	27.0/27.0	ND
191	21	ND	ND	35.0/34.4	ND
196	21	ND	ND	32.2/32.0	ND
311	21	ND	ND	29.5/30.0	ND

All samples were run in duplicate. Shown are cycle threshold (Ct) values for individual duplicate wells, separated by “/”. “–” indicates values not detected in an individual well. ND = not detected in either well. PID = post-infection day. mRPLN = medial retropharyngeal lymph node.

### Detection of SARS-CoV-2 RNA via in situ hybridization (ISH)

**Fig. 3:**
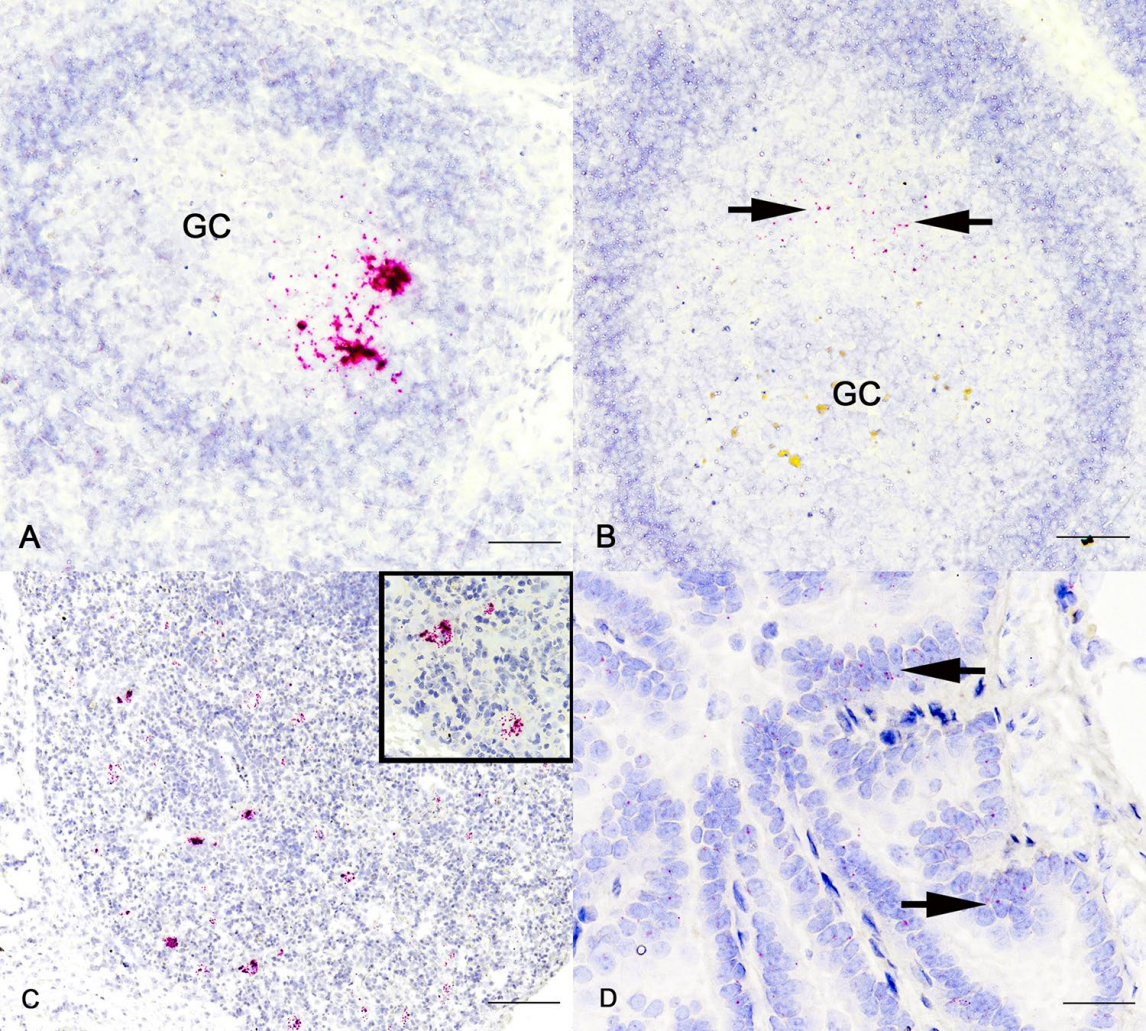
Photomicrographs of ISH labeling for SARS-CoV-2 RNA in sections of medial retropharyngeal lymph nodes (**A**; Bison #9, **B**; Bison #190) and lung (**C**; Bison #304, D; Bison #191) of bison inoculated intranasally with SARS-CoV-2 and examined 5 days (**A**, **C**) and 21 days (**B**, **D**) p.i.. Red labeling indicates presence of viral RNA. Labeling 5 days p.i. (**A**, **C**) was greater and more intense than labeling at 21 days p.i. (**B**, **D**), which was generally characterized by individual cells containing one to several dots each (arrows). Viral RNA was not detected in tissues of non-infected control bison. Bar = 200 µm (**A**, **B**, **C**), Bar = 20 µm (**D**). GC = germinal center; ISH SARS-CoV-2.

The most common tissue in which ISH revealed viral RNA was the medial retropharyngeal lymph node (Table 5). All infected bison had detectable viral RNA in 1 or more tissues. Bison examined at 2 and 5 days p.i. had labeling in a low number of germinal centers (Fig. 3A) while labeling in germinal centers of bison examined 21 days p.i. was less intense and characterized by labeling of individual cells within germinal centers (Fig. 3B). In the lung, 6 of 9 bison had labeling in one or more lung lobes examined.Labeling within the lung was most notable in areas of cellular infiltrate (Fig. 3C), as described above at 5 days p.i., as well as within alveolar, bronchial, and bronchiolar epithelial cells at all time points (Fig. 3D). No viral RNA was detectable by ISH in tissues collected from non-infected control bison.

**Table 5:** Viral RNA detection via ISH in tissues from bison collected at 2-, 5-, and 21-days p.i. with SARS-CoV-2.

Animal #	PID	Palatine tonsil	Turbinate	mRPLN	Right cranial lung	Left cranial lung	Right caudal lung	Left caudal lung
194	2	Pos	Neg	Pos	Pos	Pos	Pos	Pos
308	2	Pos	Pos	Pos	Neg	Neg	Pos	Pos
9	5	Pos	Neg	Pos	Neg	Neg	Pos	Pos
304	5	Pos	Pos	Pos	Pos	Pos	Neg	Pos
10	21	Pos	Neg	Pos	Neg	Neg	Pos	Pos
190	21	Pos	Neg	Pos	Neg	Neg	Neg	Neg
191	21	Neg	Neg	Pos	Neg	Neg	Pos	Neg
196	21	Neg	Neg	Pos	Neg	Neg	Neg	Neg
311	21	Pos	Neg	Neg	Neg	Neg	Neg	Neg

Pos, viral RNA detected by ISH; Neg, viral RNA not detected by ISH; PID, post-infection day; mRPLN, medial retropharyngeal lymph node.

### Detection of SARS-CoV-2 protein via immunohistochemistry (IHC)

**Fig. 4:**
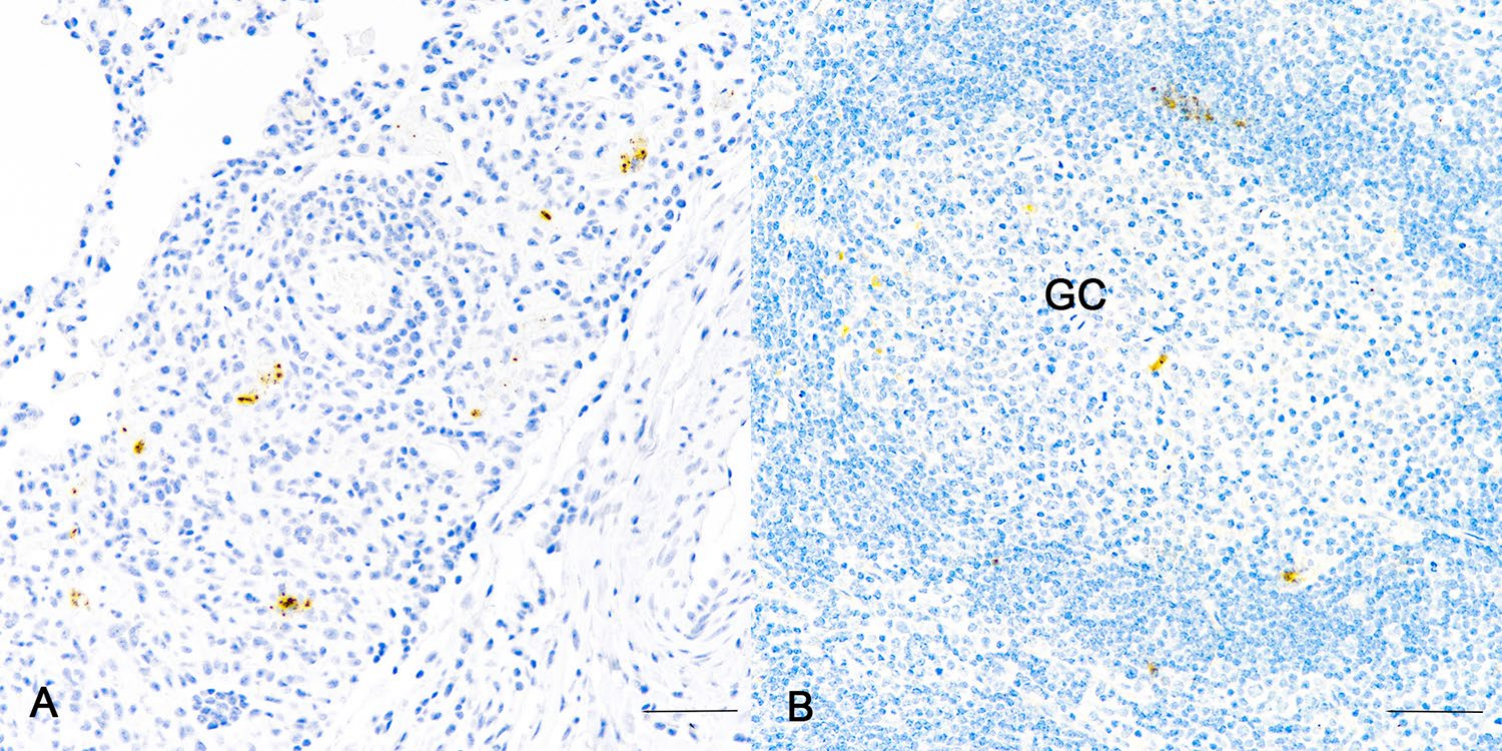
Photomicrographs of IHC labeling for SARS-CoV-2 nucleocapsid protein in lung (**A**) and medial retropharyngeal lymph node (**B**) of bison #304 inoculated intranasally with SARS-CoV-2 and examined 5 days later. Note brown labeling of nucleocapsid protein in cellular infiltrate of lung (**A**) and lymph node germinal center (GC) (**B**). No labeling was detected in tissues from non-infected control bison. Bar = 20 µm. IHC SARS-CoV-2 nucleocapsid.

Immunoreactivity to SARS-CoV-2 nucleocapsid protein was seen in a single bison (#304) examined at 5 days p.i. in the lung (Fig. 4A) amongst inflammatory cell infiltrate and in the medial retropharyngeal lymph node within and around germinal centers (Fig. 4B). No immunoreactivity was seen in bison examined at 2 or 21 days p.i. or in the other bison examined 5 days p.i. or non-infected control bison.

## Discussion

The initial outbreak of SARS-CoV-2 in a wholesale seafood market in Wuhan, China pointed to zoonotic transmission, as many cases were linked to humans with exposure to live animals sold at the market. This prompted studies to investigate the susceptibility of various animal species to infection with SARS-CoV-2 and their potential to act as sources of infection for other animals or humans. Various experimental infection studies have identified susceptible mammalian species in the Felidae, Canidae, Mustelidae, Cricetidae and Cervidae families.

In the present study, bison were moderately permissive to infection with the SARS-CoV-2 Wuhan-like variant, and developed neutralizing antibodies as measured by sVNT and VN. Moreover, viral RNA persisted in the medial retropharyngeal lymph nodes of all animals as detected by RT-qPCR, and lungs by ISH in 2 of 5 animals to the termination of the experiment at day 21 p.i. This contrasts with the minimal susceptibility of other large ruminants such as cattle in experimental settings where replication and seroconversion were limited^20,21,23,47^. More recently some surveys of cattle have demonstrated neutralizing antibody titers or viral RNA in nasal, oral or rectal swab samples suggesting a limited level of exposure and susceptibility^28,29,48^. Experimental infection studies using other domestic ruminant species such as sheep and goats have shown a low susceptibility to infection albeit with slightly greater levels of seroconversion and higher levels of viral replication in tissues than cattle^49,50^.

Gross or microscopic lesions have been absent or minimal in experimental infection studies using domestic and wild ruminants such as cattle, sheep, goats or elk^20,23,46,49–51^. Experimental infection studies using white-tailed deer resulted in microscopic pulmonary lesions with similarities to those seen in humans with COVID such as acute alveolar damage, pulmonary edema, and hyaline membranes, although ISH did not demonstrate viral RNA associated with such lesions. Bison in the current study are the first ruminant species to demonstrate lesions associated with the presence of viral RNA and/or nucleocapsid protein following experimental infection, which were seen in a single bison at 5-days post-infection.

The level of susceptibility of bison was similar to that seen in North American elk experimentally infected with the same Wuhan-like variant^46^. However, the detection of viral RNA in oronasal secretions in bison was not as great as that seen in white-tailed deer^44,45^. Similar to elk and white-tailed deer viral RNA was still detectable by RT-qPCR and ISH in retropharyngeal lymph nodes 21-days p.i.^44–46^. Unlike elk and white-tailed deer viral RNA was detectable by ISH and/or RT-qPCR in lungs of all bison examined 2- and 5- days p.i. and 2 of 5 bison examined 21-days p.i.

Persistence of viral RNA or viral proteins has been recognized in various human tissues including lymph nodes and has been proposed as an hypothesis for Long Covid^52^. Likewise, both adults and children that develop post-acute sequela of COVID-19 may not fully clear the virus and maintain viral RNA or replicating virus in various tissues including lung and lymph nodes^53^ for prolonged periods of time up to 303 days^54^. It is possible that, as with some other viruses, a reservoir of viral antigen, either RNA or protein, is present for extended periods after the acute infection and modulates the adaptive immune response^55^.

Limitations of the present study include only the presence of viral RNA was shown by RT-qPCR in swabs and tissues and does not identify replicating virus. Likewise, the probes used for ISH detected all viral RNA and were not specific for replicating virus. Additionally, as experimental infection consisted of intranasal instillation of a relatively large amount of virus, the detection of viral RNA in oronasal secretions up to 5 days p.i. could be due to residual inoculum and may not constitute true viral RNA shedding. Finally, sequencing of the inoculum after passage on Vero E6 cells revealed a mutation of the furin cleavage site of the spike protein (R682W) that has been reported as a common in vitro adaptation^57,58^. Adaptations at the furin cleavage site have been shown to limit infectivity of mammalian model hosts such as hamsters, where reduced weight loss, decreased replication in the respiratory tract and more rapid clearance have been observed compared to SARS-CoV-2 isolates with intact furin cleavage site motifs^59,60^. As such, we cannot exclude the possibility that use of an isolate lacking this mutation may have more efficiently infected bison.

There is no information on circulating SARS-CoV-2 variants in bison as no natural cases have been reported. As such we chose to use an ancestral variant, which circulated in humans early in the pandemic as it would likely be less human adapted. Results presented here demonstrate a limited level of permissiveness of bison to experimental infection with an ancestral SARS-CoV-2, evidenced by the development of virus neutralizing antibodies and persistence of viral RNA in tissues out to 21 days p.i. Although no clinical signs were seen, microscopic lesions with intralesional viral RNA in the lung suggest that infection in bison can be accompanied by limited virus induced pathology. The level of susceptibility demonstrated in the present study suggests that bison are not likely to infect other animals and are not likely to become a reservoir of infection and a source of infection for other animals or humans.

## Materials and methods

### Cells and virus

Vero E6 (ATCC® CRL-1586™) cells were cultured in Eagle’s Minimum Essential Medium (EMEM, ATCC) supplemented with 10% fetal bovine serum (FBS) and 1% antibiotic–antimycotic 100X (Gibco™, Life Technologies, Carlsbad, CA, USA). The cell cultures were maintained at 37 °C with 5% CO_2_. The lineage A SARS-CoV-2 isolate (USA-WA1/2020) was provided by BEI Resources, NIAID, NIH: SARS-Related Coronavirus 2, Isolate hCoV-19/USA-WA1/2020, NR-52281, Lot#70036318). Isolate USA-WA1/2020 originated from an oropharyngeal swab from a patient with a respiratory illness who had recently returned from travel to the affected region of China and developed clinical COVID-19 on January 19, 2020, in Washington, USA. The stock virus was passaged 3 times in Vero E6 cells, clarified by centrifugation (1000 rpm for 5 min) and stored at − 80 °C. Viral titer was determined by the Reed and Muench method^61^. A viral suspension containing 10^6^ tissue culture infectious dose 50 per ml (TCID_50_/ml) was used for inoculations.

As repeated propagation of SARS-CoV-2 on Vero E6 cells may lead to changes in the spike glycoprotein^57^, the inoculum was sequenced and compared back to the original stock from BEI resources. Two amino acid substitutions were found in the spike glycoprotein, D215G in the N-terminal domain, and R682W at the furin cleavage site.

### Animal infection and sampling

All experimental animal procedures were conducted in accordance with the recommendations in the Care and Use of Laboratory Animals of the National Institutes of Health and the Guide for the Care and Use of Agricultural Animals in Research and Teaching^62,63^. All work and procedures were approved prior to the experiment by the National Animal Disease Center (NADC) Institutional Animal and Care Use Committee (IACUC) (protocol #ARS-23–1154). All procedures and animal work were in accordance with ARRIVE guidelines and carried out in accordance with relevant guidelines and regulations.

Male bison calves (~ 6 months old; n = 9) and 3 young adult males (~ 2 years old;) were obtained from a captive herd in central Iowa, USA. Although, it would have been preferred to use bison of the same age, bison for research purposes are difficult to acquire, as such, it was necessary for us to include 3 young adult males in the study. We aimed to distribute these young males evenly, with one euthanized at 5 days p.i., one euthanized at 21 days p.i. and one assigned to the non-infected control group. All animals were sampled and screened for SARS-CoV-2 RNA by real time quantitative PCR (RT-qPCR) in oronasal secretions and by the surrogate virus neutralization test (sVNT) and virus neutralization (VN) assays prior to infection. Upon arrival (25 days prior to SARS-CoV-2 infection) all bison were treated once with tulathromycin (Draxxin; Zoetis Animal Health, Parsippany-Troy Hills, NJ, USA), injected subcutaneously at the dose recommended for cattle. Seven calves and 2 adult males were housed in an agriculture biosafety level 3 (ABSL-3) facility at the NADC and allowed to acclimate for a minimum of 2 weeks. As non-infected controls, 2 calves and 1 adult male were housed outside on pasture.

Seven calves and 2 young bulls were intranasally infected using an atomization device (LMA MAD Nasal™, Teleflex; Morrisville, NC, USA) delivering approximately 2.5 ml of inoculum into each nostril for a total of 5 ml. On days 0, 2, 3, 4, 5, 7, 10, 14 and 21 post-infection (p.i.) nasal, oral and rectal swabs were collected for RT-qPCR. Blood was collected on days 0, 7, 14 and 21 days p.i. for serologic assays. For sampling, manual restraint in a squeeze chute, without chemical restraint was used.

### Serology

The cPASS SARS-CoV-2 neutralization antibody detection kit (GenScript Biotech,

Amsterdam, Netherlands) was used as described^64,65^ and according to the manufacturer’s recommendations. The assay detects the presence of specific anti-SARS-CoV-2 neutralizing antibodies against the S-protein in serum of all species and in an isotype-independent manner by blocking the interaction between the RBD of the viral spike glycoprotein with the ACE2 cell surface receptor. The absorbance at 450 nm of the sample is inversely dependent on the titer of the anti-SARS-CoV-2 neutralizing antibodies in tested samples. To confirm the sVNT results, serum samples were submitted to the Animal Health Diagnostic Center, College of Veterinary Medicine, Cornell University, Ithaca, NY, USA for VN testing.

### Necropsy and sample collection

Animals were humanely euthanized by sedation with a combination of intravenous xylazine and ketamine followed by intravenous pentobarbital sodium to achieve euthanasia. Two inoculated calves and an inoculated calf and young bull were euthanized on days 2 and 5 p.i., respectively, while the remaining animals were euthanized on day 21 p.i.. Similarly, non-infected controls were euthanized and examined at the termination of the study. Following euthanasia, multiple tissues (palatine tonsil, nasal turbinate, medial retropharyngeal, tracheobronchial and mediastinal lymph nodes, lung (right and left caudal lobes), heart, liver, spleen, and kidney were collected. Samples were individually bagged, placed on dry ice, and transferred to a -80 °C freezer until testing by RT-qPCR. The tissues listed above plus samples from 4 lung lobes (right and left cranial, right and left caudal) were also collected and processed for standard microscopic examination. A subset of tissues, based on prior experience with experimental infection of cattle, elk, and white-tailed deer, was also processed by in situ hybridization (ISH) and immunohistochemistry (IHC). For this, tissue sections of approximately ≤ 0.5 cm in width were fixed by immersion in 10% neutral buffered formalin (≥ 20 volumes fixative to 1 volume tissue) for approximately 24 h, and then transferred to 70% ethanol, followed by standard paraffin embedding techniques. Slides for standard microscopic examination were stained with hematoxylin and eosin (HE). Adjacent or near adjacent unstained sections were used for ISH and IHC. Slides were examined by light microscopy using a Nikon Eclipse Ci microscope (Nikon Instruments Inc, Melville, NY, USA) equipped with a Nikon DS-Ri2 digital camera (Nikon Instruments Inc). Images were captured using Nikon Elements D software (Nikon Instruments, Inc). Images were sized and annotated using Adobe Photoshop 2025 (Adobe, San Jose, CA, USA).

### Real-time quantitative RT-qPCR on swabs and tissues

To assess viral RNA on nasal, oral and rectal samples, swabs were submitted to the National Wildlife Research Center, Ft. Collins, CO, USA for RT-qPCR analysis. To determine the presence of viral RNA in tissue samples, tissues were thawed, cut into an approximately 50–100 mg piece, and resuspended in 1–2 mL of TRI-Reagent® (Life Technologies, Carlsbad, CA, USA) in individual gentle MACS™ M tubes (Miltenyi Biotec, Bergisch Gladbach, Germany). Tissues were dissociated using a gentle MACS™ Octo-Dissociator (Miltenyi Biotec) following the manufacturer’s recommendations. RNA was extracted from tissue homogenate samples using the MagMAX™-96 for Microarrays Total RNA Isolation Kit (Applied Biosystems, Waltham, MA, USA). Samples were run on a MagMAX™ Express Magnetic Particle Processor (Applied Biosystems) following the manufacturer’s instructions. Next, 15 µl of extracted product was added to 5 µl of the AgPath-ID™ One step RT-qPCR master mix (Applied Biosystems). Samples were run in duplicate, The RT-qPCR reactions were performed on an ABI 7500 Fast instrument (Applied Biosystems) run in standard mode with the following conditions: 1 cycle at 45 °C for 10 min, followed by 1 cycle at 95 °C for 10 min, 1 cycle at 95 °C for 3 s, and 45 cycles at 55 °C for 30 s. The forward primer sequence was 5′-GACCCCAAAATCAGCGAAAT-3′, the reverse primer sequence was 5′-TCTGGTTACTGCCAGTTGAATCTG-3’, and the probe sequence was 5′-FAM-ACCCCGCATTACGTTTGGTGGACC-BHQ1-3’. A positive control (2019-nCoV_N_Positive Control, Integrated DNA Technologies IDT, Coralville, IA, USA) and a negative control were run on every plate.

### In situ hybridization (ISH)

Paraffin-embedded tissues were sectioned at 5 µm and subjected to ISH using the RNAscope ZZ probe technology (Advanced Cell Diagnostics, Newark, CA). In situ hybridization was performed to visually detect tissue distribution of SARS-CoV-2 RNA in tissues. Nasal turbinate, palatine tonsil, medial retropharyngeal lymph node, and lung were tested using the RNAscope 2.5 HD Reagents–RED kit (Advanced Cell Diagnostics) as previously described^45^. Proprietary ZZ probes targeting SARS-CoV-2 RNA (V-nCoV2019-S probe Cat # 848561) were used for detection of viral RNA. A positive control probe targeted the *Bos taurus* –specific cyclophilin B (PPIB Cat # 3194510) or ubiquitin (UBC Cat # 464851) housekeeping genes, while a probe targeting dapB of *Bacillus subtilis* (Cat # 312038) was used as a negative control.

### Immunohistochemistry

Immunohistochemical staining for SARS-CoV-2 was performed on sections of palatine tonsil, nasal turbinate, medial retropharyngeal lymph node, and lung. Formalin-fixed paraffin-embedded tissues were prepared for staining by baking at 57 °C for 45 min. Tissues were then deparaffinized with xylene and rehydrated through a series of graded alcohol solutions. To perform epitope retrieval, slides were submerged in a 1X citrate unmasking solution (Antigen Retrieval Buffer; Abcam, Cambridge, United Kingdom) until boiling was initiated and then maintained in the unmasking solution at a sub-boiling temperature (95–98 °C) for 10 min. A 3% hydrogen peroxide solution (Fisher Bioreagents, Waltham, MA, USA) was used to quench endogenous peroxidases. Slides were then immersed in a blocking solution of Tris Buffered Saline (Thermo Fisher, Waltham, MA, USA) and Tween20® (Sigma-Aldrich; St Louis, MO, USA) with 5% normalized goat serum. A rabbit monoclonal antibody targeting the SARS-CoV-2 Nucleocapsid Protein (HL344) at a concentration of 1:800 was used as the primary antibody (Cell Signaling Technologies, Boston, MA, USA). Tissues were then incubated in SignalStain® Boost IHC Detection Reagent (HRP, Rabbit, Cell Signaling Technologies) followed by SignalStain® 3,3’ diaminobenzidine (DAB) substrate to produce a brown reaction product (Cell Signaling Technologies). Finally, counterstaining was performed using hematoxylin stain solution and Bluing Reagent (Ventana Medical Systems; Santa Clara, CA, USA). Sections of nasal turbinate tissue from a single American mink (*Neovison vison*) inoculated with SARS-CoV-2 served as positive control samples.

## Declarations

### Data availability

The genome of the SARS-CoV-2 strain used in this study is available from NCBI (https://www.ncbi.nlm.nih.gov/), accession PV714139.
